# Endemic vascular plants in the Ukrainian Carpathians

**DOI:** 10.3897/BDJ.10.e95910

**Published:** 2022-12-13

**Authors:** Andriy Novikov, Mariia Sup-Novikova

**Affiliations:** 1 State Museum of Natural History of the NAS of Ukraine, Lviv, Ukraine State Museum of Natural History of the NAS of Ukraine Lviv Ukraine; 2 Ukrainian Catholic University, Lviv, Ukraine Ukrainian Catholic University Lviv Ukraine

**Keywords:** occurrence, herbarium material, chorology, mountain flora

## Abstract

**Background:**

This dynamic dataset aims to gather all available data, extracted mostly from the preserved material deposited at the leading Ukrainian herbaria on the distribution of the endemic vascular plants in the Ukrainian Carpathians and adjacent territories. This dataset is created in the framework of mapping the distribution of the endemic plants and is aimed to unveil the patterns of their spatial distribution, ecological preferences and temporal trends in the flora of the Ukrainian Carpathians. A total of 76 species and subspecies of vascular plants belonging to 49 genera and 27 families are reported herein to occur in the Ukrainian Carpathians and close regions. Amongst the total number of reported 6,427 occurrence records, 1,961 records are georeferenced and supported with a translation of Cyrillic information into English. The remaining occurrence records will be georeferenced and translated into English in the near future, as well as the dataset being completed with new records obtained from the new sources.

**New information:**

In total, 6,427 occurrence records of the endemic vascular plants distributed in the Ukrainian Carpathians were published.

## Introduction

For years, the data about plant diversity in Ukraine were mostly published in Cyrillic languages (mostly Ukrainian and Russian) in local journals that are hard to find and read for foreign researchers. Simultaneously, most of the collected plant materials are deposited in the local Ukrainian herbaria that are complicated to access for different reasons (Novikov 2019). In 2020-2021, amongst the challenges for researchers working with herbarium collections globally were travel limitations caused by the pandemic situation (Baker 2020, Baldini 2020). However, in 2022, the war with Russia made natural history collections and herbaria, in particular, in Ukraine unavailable and catastrophically increased the risk of their damage and loss. The loss of specimens of Red-listed and endemic plant taxa deposited in the Ukrainian herbaria will be irreparable because some of them are already extinct in the flora of Ukraine and some were collected only occasionally many years before. The loss of such specimens can also corrupt the data on changes in the spatial distribution patterns of these taxa. Therefore, the actuality of databasing and digitisation of the Ukrainian herbaria and related information appears a crucial modern task providing a number of benefits for scientists worldwide, including fast and easy access, translation from local languages into English, long-term virtual preservation of specimens, crosslinking of specimens and collectors data etc. (Holovachov et al. 2014, Cantrill 2018, Hedrick et al. 2020).

The Ukrainian Carpathians Mts. represent one of the leading centres of biodiversity in Ukraine, with numerous endemic, rare and relict plant species. In particular, there occur 2532 species of vascular plants, which comprise near 49% of the entire flora of Ukraine (Mosyakin and Fedoronchuk 1999, Chopyk and Fedoronchuk 2015). Of this number, about 70 plant species and infraspecific taxa are endemic (Novikoff and Hurdu 2015) and 207 are listed in the Red Book of Ukraine (Didukh 2009, Didukh 2010). Taking into account the extreme value of the endemic plants for biodiversity investigations (see Casazza et al. 2008, Cañadas et al. 2014, Harrison and Noss 2017 for examples), the main aim of the current dataset was to provide free and open access to the data on distribution of the endemic vascular plants in the Ukrainian Carpathians.

The compilation of this dataset began in 2015. Since then, it has been continuously updated, based on the elaboration of new collections and publications, providing georeferenced and translated data from the verbatim herbarium labels and occurrences' reports.

## General description

### Purpose

The purpose of creating an online publication of this dataset is to secure the future of the Ukrainian collections and related data on endemic vascular plants distributed in the Ukrainian Carpathians. Making this dataset freely accessible and digitally available through GBIF ensures its virtual preservation and survival in case of the involved collections' loss or damage. Moreover, this also pursues wider application of biodiversity data from Ukraine in international research projects, allowing the white spots on the world map to be covered.

## Sampling methods

### Study extent

The dataset contains information on 6,427 occurrences of endemic vascular plants from the Ukrainian Carpathians and some adjacent Ukrainian territories (Novikov and Sup-Novikova 2022).

### Sampling description

Initially, 70 endemic and subendemic (i.e. mainly distributed in the Carpathian Mts., although with few occurrences outside their limits – Kliment et al. 2016, Mráz et al. 2016) species and subspecies of vascular plants were selected for the analysis (Novikoff and Hurdu 2015). Later, the initial list was critically revised, based on an analysis of protologues, distribution reports, other newly-available published sources and elaboration of herbarium material. As a result, the final checklist contains 76 taxa with 946 synonyms that were used for work at the herbaria and search of occurrences' reports in the publications. For published occurrences, we selected only those provided by reliable authors (e.g. well-known florists) in peer-reviewed journals and supported with coordinates or precise locality descriptions. The occurrences reported by Karel Domin in his Card Index, deposited at the Institute of Botany of SAS in Bratislava, also were partly taken into account, with the exception of doubtful or unclear indications of localities. Due to the high risk of misidentification of problematic taxa, we avoid clearly doubtful reports from persons without recognised expertise in botany.

### Quality control

The gathered data were double-checked for the correctness of identification and indicated distribution area. In case of doubt or misidentification, specimens were re-identified or omitted from the analysis. In case of unusual reports from the new areas, such occurrences were additionally critically revised. In the case of identification of problematic taxa, special attention was paid to those specimens identified by narrow specialists only. Most of microtaxa (e.g. *Hieracium*, *Alchemilla* and *Pilosella*) and some stenoendemics were excluded from the work due to a high risk of their misidentification, unclear chorology and controversial taxonomical interpretation by different authors. The taxonomy, with minor exceptions, was verified and follows the POWO checklist (POWO 2022). Coordinates of the occurrences were extracted and verified manually, using the OpenStreetMap (OpenStreetMap contributors 2022) and QGIS (QGIS Development Team 2022) services.

### Step description

The following steps were taken during the work with herbarium materials:

1. Routine photo capture of herbarium labels for selected taxa;
2. Taxonomic reconsideration of photographed specimens following recent taxonomy;
3. Extracting the locality, collector, date and other relevant (e.g. habitat information, identification history) information from the labels to the dataset using the DarwinCore standard;
4. Translation of the initial information from Cyrillic (i.e. Ukrainian and Russian) into English;
5. Georeferencing and verification of localities using recent and antique maps;
6. Quality check applying OpenRefine and QGIS (for outlets and coordinates' mistakes).

The following steps were taken during the work with literature reports:

1. Verification of the report authors on their authority;
2. Manual extraction of published data to the dataset following the DarwinCore standard;
3. Translation of the initial information from Cyrillic into English;
4. Georeferencing and verification of localities;
5. Data quality control.

## Geographic coverage

### Description

**Figure 1.**
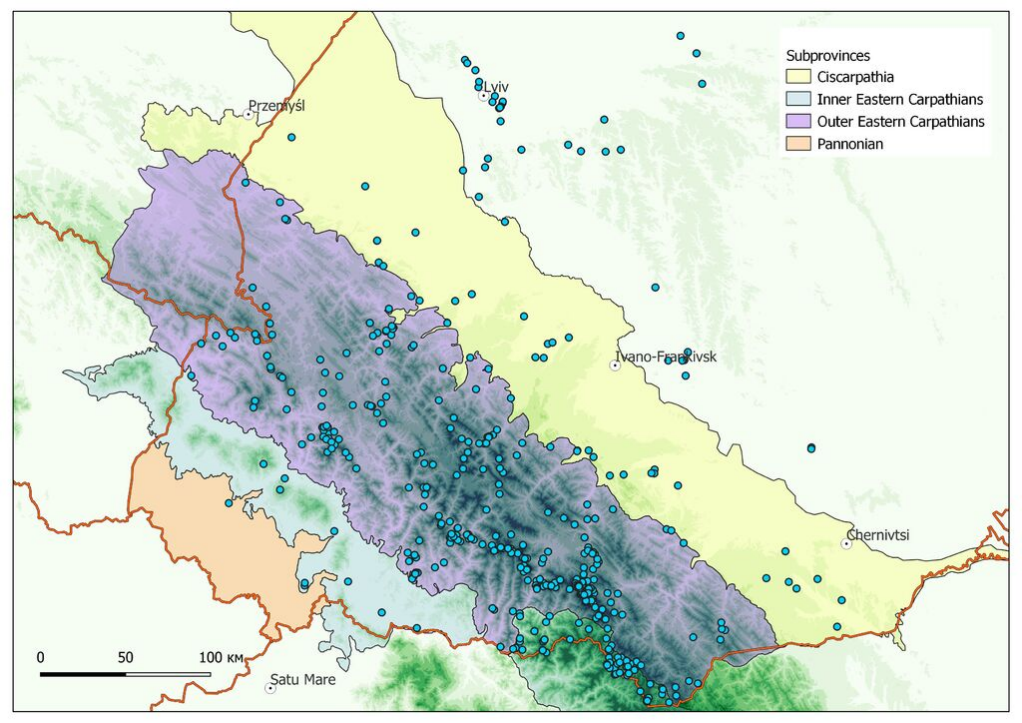
Distribution of georeferenced occurrences of the endemic vascular plants in the Ukrainian Carpathians and adjacent territories.

The occurrences from the Ukrainian Carpathians and adjacent Ukrainian territories were considered (Fig. 1). Most of the covered occurrences rely on the territory of Ukrainian Carpathian Mts. However, some taxa (e.g. *Symphytumcordatum*, *Aconitummoldavicum* and *Cardamineglanduligera*) are spread widely out of the Carpathians and can be found in adjacent regions. To represent the real distribution patterns of such subendemic taxa, their findings from the non-Carpathian regions were also taken into consideration.

### Coordinates

47.8 and 51.5 Latitude; 22.7 and 25.9 Longitude.

## Taxonomic coverage

### Description

**Figure 2.**
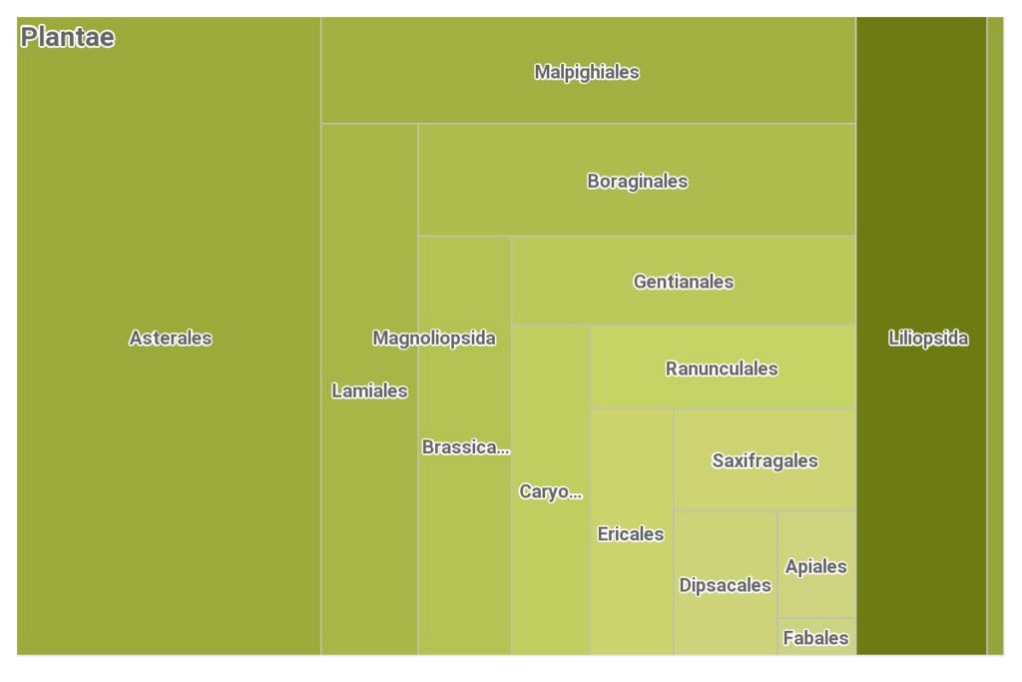
Taxonomic coverage of the dataset.

All processed occurrences were identified to the level of the species or subspecies. As a result, the dataset contains 66 species and subspecies of endemic and subendemic vascular plants belonging to 49 genera and 27 families. Amongst them, only 17 species from 10 genera (i.e. 967 occurrences or 15% from the total number of databased occurrences) represent the class Liliopsida, while the rest of the taxa belongs to the class Magnoliopsida (Fig. 2).

### Taxa included

**Table:** 

Rank	Scientific Name	
kingdom	Plantae	
phylum	Tracheophyta	
class	Magnoliopsida	
class	Liliopsida	
order	Gentianales	
order	Asparagales	
order	Saxifragales	
order	Poales	
order	Asterales	
order	Fabales	
order	Lamiales	
order	Brassicales	
order	Caryophyllales	
order	Apiales	
order	Gentianales	
order	Dipsacales	
order	Ericales	
order	Ranunculales	
order	Boraginales	
order	Malpighiales	
family	Rubiaceae	
family	Orchidaceae	
family	Saxifragaceae	
family	Poaceae	
family	Asteraceae	
family	Fabaceae	
family	Iridaceae	
family	Campanulaceae	
family	Plantaginaceae	
family	Brassicaceae	
family	Caryophyllaceae	
family	Asparagaceae	
family	Lamiaceae	
family	Apiaceae	
family	Gentianaceae	
family	Crassulaceae	
family	Caprifoliaceae	
family	Oleaceae	
family	Juncaceae	
family	Rubiaceae	
family	Primulaceae	
family	Orobanchaceae	
family	Ranunculaceae	
family	Boraginaceae	
family	Violaceae	
family	Salicaceae	
family	Linaceae	
genus	*Galium*	
genus	*Gymnadenia*	
genus	*Chrysosplenium*	
genus	*Sesleria*	
genus	*Saussurea*	
genus	*Lathyrus*	
genus	*Crocus*	
genus	*Phyteuma*	
genus	*Plantago*	
genus	*Campanula*	
genus	*Noccaea*	
genus	*Antennaria*	
genus	*Koeleria*	
genus	*Silene*	
genus	*Doronicum*	
genus	*Scilla*	
genus	*Thymus*	
genus	*Achillea*	
genus	*Poa*	
genus	*Festuca*	
genus	*Heracleum*	
genus	*Gentiana*	
genus	*Cardamine*	
genus	*Leucanthemum*	
genus	*Trifolium*	
genus	*Sempervivum*	
genus	*Scabiosa*	
genus	*Minuartia*	
genus	*Syringa*	
genus	*Luzula*	
genus	*Sabulina*	
genus	*Galium*	
genus	*Genista*	
genus	*Erysimum*	
genus	*Soldanella*	
genus	*Melampyrum*	
genus	*Arabidopsis*	
genus	*Alopecurus*	
genus	*Ranunculus*	
genus	*Pulmonaria*	
genus	*Viola*	
genus	*Scorzoneroides*	
genus	*Centaurea*	
genus	*Swertia*	
genus	*Trisetum*	
genus	*Euphrasia*	
genus	*Salix*	
genus	*Linum*	
genus	*Symphytum*	
species	*Galiumalbum*	
species	*Gymnadeniacarpatica*	
species	*Chrysospleniumalpinum*	
species	*Sesleriabielzii*	
species	*Saussureaporcii*	
species	*Lathyrustranssylvanicus*	
species	*Crocusbanaticus*	
species	*Phyteumavagneri*	
subspecies	Plantagoatratasubsp.carpatica	
species	*Campanulaserrata*	
species	*Sesleriaheufleriana*	
species	*Noccaeadacica*	
species	*Antennariacarpatica*	
subspecies	Koeleriamacranthasubsp.transsilvanica	
species	*Silenezawadskii*	
species	*Doronicumcarpaticum*	
species	*Scillakladnii*	
species	*Thymuspulcherrimus*	
subspecies	Achilleaoxylobasubsp.schurii	
species	*Poarehmannii*	
species	*Festucaporcii*	
species	*Heracleumcarpaticum*	
species	*Gentianalaciniata*	
species	*Cardamineglanduligera*	
species	*Leucanthemumrotundifolium*	
species	*Thymusalternans*	
species	*Trifoliumsarosiense*	
subspecies	Sempervivumcarpathicumsubsp.carpathicum	
subspecies	Poapannonicasubsp.scabra	
species	*Scabiosalucida*	
subspecies	Sempervivumglobiferumsubsp.preissianum	
species	*Minuartiapauciflora*	
species	*Syringajosikaea*	
species	*Festucaversicolor*	
subspecies	Luzulaalpinopilosasubsp.obscura	
species	*Campanulacarpatica*	
species	*Sabulinaoxypetala*	
species	*Festucacarpatica*	
species	*Galiumtranscarpaticum*	
subspecies	Genistatinctoriasubsp.oligosperma	
species	*Erysimumwitmannii*	
species	*Campanulatatrae*	
species	*Soldanellahungarica*	
species	*Melampyrumsaxosum*	
subspecies	Festucaamethystinasubsp.orientalis	
species	*Poacarpatica*	
species	*Arabidopsisneglecta*	
subspecies	Alopecuruspratensissubsp.laguriformis	
species	*Ranunculuscarpaticus*	
subspecies	Heracleumsphondyliumsubsp.carpaticum	
species	*Phyteumatetramerum*	
species	*Pulmonariafilarszkyana*	
species	*Violadeclinata*	
species	*Scorzoneroidespseudotaraxaci*	
species	*Centaureamaramarosiensis*	
subspecies	Poagraniticasubsp.disparilis	
species	*Silenedubia*	
species	*Swertiaperennis*	
subspecies	Centaureaphrygiasubsp.carpatica	
species	*Trisetumfuscum*	
species	*Erysimumwitmannii*	
species	*Euphrasiatatrae*	
subspecies	Salixretusasubsp.kitaibeliana	
species	*Linumextraaxillare*	
species	*Ranunculusmalinovskii*	
species	*Symphytumcordatum*	

## Temporal coverage

**Living time period:** 1852-2020.

### Notes

**Figure 3.**
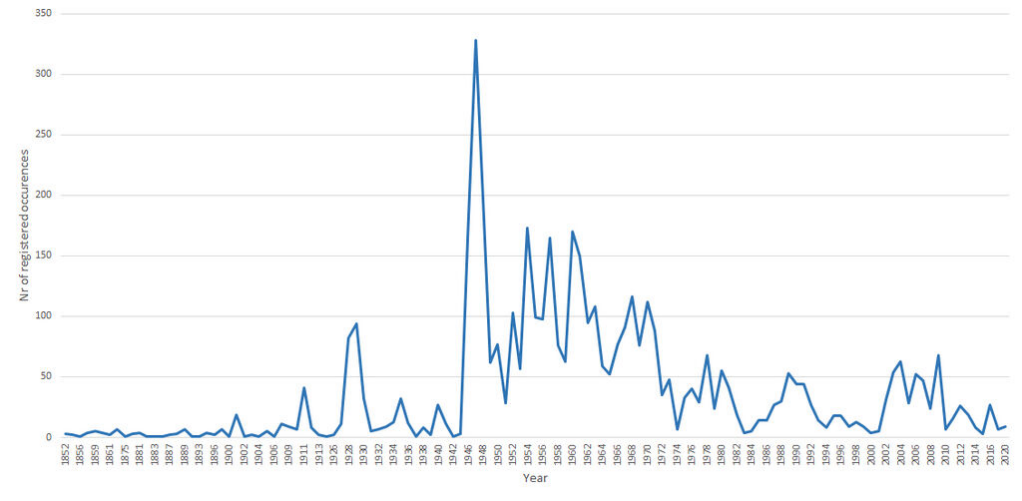
The number of registered occurrences per year in the dataset.

The dataset covers material collected and reported from 1852 till 2020. However, the most intensive records were made in the period between 1945 and 1975. During these thirty years, 3,013 or ca. 47% from the total number of observations were made (Fig. 3).

## Usage licence

### Usage licence

Open Data Commons Attribution License

## Data resources

### Data package title

Endemic vascular plants of the Ukrainian Carpathians

### Resource link

https://www.gbif.org/dataset/f14ffffd-5fd1-440d-a4c5-07ddce62ff26

### Alternative identifiers

https://doi.org/10.15468/5hrh87

### Number of data sets

1

### Data set 1.

#### Data set name

Endemic vascular plants of the Ukrainian Carpathians

#### Data format

Darwin Core

#### Character set

utf8

#### Download URL

https://www.gbif.org/dataset/f14ffffd-5fd1-440d-a4c5-07ddce62ff26

#### Description

The tab-delimited CSV formated dataset (Novikov and Sup-Novikova 2022) is created with the application of Darwin Core standards and contains all available data on the distribution of endemic vascular plants in the Ukrainian Carpathians and adjacent territories.

**Data set 1.** 

Column label	Column description
occurrenceID	An unique identifier for the Occurrence (as opposed to a particular digital record of the occurrence).
basisOfRecord	The specific nature of the data record, for example, preserved specimen or field observation.
collectionCode	Unique code of collection (e.g. herbarium) for depositing the identified specimen.
catalogNumber	An identifier for the record within the collection.
scientificName	The full scientific name of the taxon including at least the genus and species epithets and, in some cases, including the subspecies epithet.
taxonRank	The taxonomic rank of the most specific name in the scientificName.
recordedBy	A person, group or organisation responsible for recording the original Occurrence.
verbatimEventDate	The date of record as it appears in the original publication or specimen's label.
EventDate	The date during which an event (e.g. collection of the specimen, photographing of the plant or its registering in the field in any other way), occurred.
day	The day when occurrence was recorded.
month	The month when occurrence was recorded.
year	The year when occurrence was recorded.
fieldNumber	An identifier given to the specimen in the field by the collector.
identifiedBy	A list of names of people, who assigned the Taxon to the subject.
dateIdentified	The date on which the subject was determined as representing the Taxon.
identificationRemarks	Comments or notes about the Identification.
decimalLatitude	The geographic latitude (in decimal degrees, using the spatial reference system given in geodeticDatum) of the geographic centre of a Location.
decimalLongitude	The geographic longitude (in decimal degrees, using the spatial reference system given in geodeticDatum) of the geographic centre of a Location.
coordinateUncertaintyInMetres	The horizontal distance (in metres) from the given decimalLatitude and decimalLongitude describing the smallest circle containing the whole of the Location.
geodeticDatum	The ellipsoid, geodetic datum or spatial reference system (SRS) upon which the geographic coordinates given in decimalLatitude and decimalLongitude are based. In our case, it is always WGS84.
verbatimElevation	The original description of the elevation (altitude, usually above sea level) of the Location.
countryCode	The standard code (ISO 3166-1-alpha-2) for the country in which the Location occurs.
country	The name of the country in which the Location occurs.
locality	The specific description of the place where the specimen was registered or collected.
verbatimLocality	The original textual description of the place where the specimen was registered or collected.
fieldNotes	The original text of notes taken in the field about the specimen by the collector.
associatedReferences	A list (concatenated and separated) of identifiers (publication, bibliographic reference, global unique identifier, URI) of literature associated with the Occurrence.
kingdom	The full scientific name of the kingdom in which the taxon is classified. In our case, it is always Plantae.
language	The language of the resource. In our case, herbarium labels contained information in different languages and sometimes different languages were even combined on a single label. To simplify the work with data, we indicated the languages applied for the data.
minimumElevationInMetres	The lower limit of the range of elevation (altitude, usually above sea level), in metres.
maximumElevationInMetres	The upper limit of the range of elevation (altitude, usually above sea level), in metres.
institutionCode	The acronym in use by the institution having custody of the object(s) or information referred to in the record. We followed the GBIF register of institutions and collections for acronyms.

## Additional information

This dataset does not include the data on distribution of the genus *Aconitum* representatives because such data were published before, in 2021 and available as a separate dataset (Novikov 2021). We decided not to include the data on *Aconitum* distribution to avoid duplication of the same records.
